# Supplementary material for: Homogeneity of agriculture landscape promotes insecticide resistance in the ground beetle Poecilus cupreus
Source: PLoS One. 2022 Apr 26;17(4):e0266453. doi: 10.1371/journal.pone.0266453 (PMC9041758; doi:10.1371/journal.pone.0266453)
Supplement: S1 Table — (DOCX) [file pone.0266453.s001.docx]

SUPPLEMENTARY MATERIAL FOR:

**Full title**: Homogeneity of agriculture landscape promotes insecticide resistance in the ground beetle *Poecilus cupreus*

**Short title: Agriculture landsapce simplification promotes insecticide resistance**

Grzegorz Sowa^1*^, Agnieszka J. Bednarska^2^, Elżbieta Ziółkowska^1^, Ryszard Laskowski^1^

^1^Institute of Environmental Sciences, Jagiellonian University, Kraków, Poland

^2^Institute of Nature Conservation, Polish Academy of Sciences, Kraków, Poland

* Corresponding author

e-mail: grzegorz.sowa@uj.edu.pl (GS)

**Table S1. PROTEUS 110 OD (Bayer, Germany) specification**

| Name | CAS-No. /  EC-No. /  REACH Reg. No. | Concentration [%] |
| --- | --- | --- |
| Thiacloprid | 111988-49-9 | 10.0 |
| Deltamethrin | 52918-63-5  258-256-6 | 1.0 |
| 2-Ethylhexanol propylene  ethyleneglycol ether | 64366-70-7 | > 1 – < 25 |
| Dodecyl benzene  sulphonate, calcium salt | 26264-06-2  247-557-8 | > 5 – < 10 |
| 2-Ethylhexanole | 104-76-7  203-234-3 | > 1 – < 20 |
| Alcohols, C12-C15-  branched and linear,  ethoxylated | 106232-83-1  500-294-5 | > 1 – < 5 |
| 2,6-Di-tert-butyl-4-  methylphenol | 128-37-0  204-881-4  01-2119555270-46-xxxx | > 0.1 – < 1 |
