# Supplementary material for: Homogeneity of agriculture landscape promotes insecticide resistance in the ground beetle Poecilus cupreus
Source: PLoS One. 2022 Apr 26;17(4):e0266453. doi: 10.1371/journal.pone.0266453 (PMC9041758; doi:10.1371/journal.pone.0266453)
Supplement: Attachment — Submitted filename: Response_to_Reviewer_PONE-D-21-27829_R2.pdf [file pone.0266453.s004.pdf]

Response to the Reviewer (R2)

We acknowledge receiving the decision rejecting our manuscript based on the review, but we fundamentally do not agree with the opinion expressed by the Reviewer and, consequently, the decision made by the Academic Editor. The Reviewer had only one comment, relating to the use of terms “tolerance” and “resistance” in our manuscript. Below please find the comment itself (in blue italics, indented) followed by our rebuttal.

> *Reviewer #1: # PONE-D-21-27829R2*
>
> *Title: Homogeneity of agriculture landscape promotes insecticide resistance in the ground beetle Poecilus cupreus*
>
> *Brief.*
>
> *The manuscript was partially revised following suggestions from the reviewers. Furthermore, even after revising the terms used in the manuscript, the author chose to keep the wrong term and justify the error by reediting the term used in another manuscript.*
>
> *General comments*
>
> *The comments about lines 339 and 351 and 360 to 362 is not suggesting any discuss. The terms “tolerance” and “resistance” in results and discussion section was not clear and it remain unclear. Briefly, resistance may be defined as “a heritable change in the sensitivity of a pest population that is reflected in the repeated failure of a product to achieve the expected level of control when used according to the label recommendation for that pest species” (IRAC, 2022 [https://irac-online.org/about/resistance/]). In contrast to resistance, insecticide tolerance is a natural tendency and not a result of selection pressure and following Tabashnik (1991) insecticide resistance can be viewed as a threshold trait, with tolerance as the underlying continuous variable (Finney 1971, Falconer 1981, Via 1986, Tabashnik & Cushing 1989). Thus, the term should be rethought, because if beetles have been exposed to active ingredients at the collect site and exposed in the generations P, F1 and F2 generations, would they be resistant or tolerant? There is an expectation supported by results found that beetles are not tolerant, but individuals resistant. This would all change the way approach this term in the sections. Again, a discussion of this in any section is not recommended.*
>
> *Bruce E. Tabashnik, 1991. Deterrnining the Mode of Inheritance of Pesticide Resistance with Backcross Experiments. Journal of Economic Entomology. V. 84, no. 3, p.703-712.*
>
> *The explanation provide by authors of manuscript about lines 408 to 409 in the response to reviewers is completely wrong and they are reediting the terms “susceptibility and resistance” used correctly by Schoonees and Giliomee (1982).*

We do not agree with the opinion expressed by the Reviewer and with the decision of the Academic Editor rejecting our manuscript. First of all, it should be noted that the Reviewer did not indicate any flaws in our study, data analysis or the manuscript itself except of contesting the use of just two terms: “tolerance” and “resistance”. In our opinion this might be, at the very best, the subject of discussion on the semantics but does not justify rejecting

the manuscript. The questioned terms do not have such a strict scientific meaning as the Reviewer tries to imply. The meaning of the terms is defined in different ways, depending on the need, as shown in the examples below. Although some authors can define them in a way indicated by the Reviewer, this does not mean that such an understanding is the only one and obligatory. More importantly, in the revised version and in our previous Response to the Reviewer we explained very clearly how both terms are understood (defined) in our manuscript and why we decided to use both. Briefly, by testing *tolerance* to a pesticide (by estimating LC50 and comparing survival curves) throughout three generations we could conclude that most probably some of the tested populations exhibited genetically fixed *resistance*. This is exactly in accordance with the definition proposed by Tabashnik and Johnson in the "Handbook of Biological Control" (by the way, one of the authors, B. E. Tabashnik, is the very same author as the one cited by the Reviewer!):

Examples of the use of terms "resistance" and "tolerance"

"*Pesticide **resistance** is a genetically based, statistically significant increase in the ability of a population to **tolerate** one or more pesticides. In most cases, resistance is documented with laboratory bioassays showing that a population with a history of extensive exposure to pesticides has a significantly greater LC50 or LD50 [...] compared with a conspecific population that has had less exposure to pesticides. One can also document resistance by showing that treatment with a fixed concentration or dose causes significant differences in mortality among conspecific populations [...]. Evidence of significant increase through time within a population in LC50, LD50, or survival in response to a fixed concentration or dose provides more direct documentation of resistance."* (from "Evolution of Pesticide Resistance in Natural Enemies" by B. E. Tabashnik, M. W. Johnson, in "Handbook of Biological Control", 1999).

In turn, EPA defines tolerance in a very precise way for a very specific purpose: "*A tolerance is the EPA established maximum residue level of a specific pesticide chemical that is permitted in or on a specific human or animal food in the United States*".

In pharmacology "*Tolerance is a decrease in response to a drug that is used repeatedly. Resistance is development of the ability to withstand the previously destructive effect of a drug by microorganisms or tumor cells*".

Referring to pesticide resistance and tolerance: "*The chemical arsenal we have developed in an attempt to rid our homes of rodents and our crops of insects is losing its power. We have simply caused pest populations to evolve, unintentionally applying artificial selection in the form of pesticides. Individuals with a higher tolerance for our poisons survive and breed, and soon resistant individuals outnumber the ones we can control.*" (https://www.pbs.org/wgbh/evolution/library/10/1/l_101_02.html)

Please note also that the English language thesaurus reports "resistance" and "resilience" (together with "strength" and "toughness") as the closest synonyms of "tolerance".

Moreover, the Reviewer's statement about the definition of resistance ("*resistance may be defined as "a heritable change in the sensitivity of a pest population that is reflected in the repeated failure of a product to achieve the expected level of control when used according to the label recommendation for that pest species*") is completely irrelevant to our studies as we did not study effectiveness of any product against pests but rather the combined effect of in-field pesticide applications and landscape structure on the resistance of a beneficial carabid.

In our earlier response to the Reviewer, we provided a detailed answer to the Reviewer's comments and explained that only "because similar results of increased *tolerance* to the insecticide were found in beetles from the LM and LL habitats over three generations (P, F1 and F2), the results suggest the evolution of *resistance*" (in terms of *a heritable change in the sensitivity*), as by doing the study on three generations of beetles we could "separate the actual genetically fixed adaptation (=heritable *resistance*) from possible direct or maternal effects". We also explained that the increased *tolerance* to an insecticide means the *decreased sensitivity* to that insecticide. Even if this is not exactly the same as the Reviewer's reasoning, we are convinced that this is, at the best, a field for polemics rather than a basis for rejecting the manuscript. Indeed, if the Editor prefers to have the term "less sensitive" instead of "tolerant" (although *tolerant* means simply *less sensitive*), we do not see a problem with such a rewording.

We completely do not agree with the Reviewer's statement that "*The explanation provide by authors of manuscript about lines 408 to 409 in the response to reviewers is completely wrong and they are reediting the terms "susceptibility and resistance" used correctly by Schoonees and Giliomee (1982).*" We read the paper carefully and would like to know where exactly we distorted the meaning of the words.

It must be stressed that the decision to reject our manuscript was based on the opinion of just one reviewer who apparently did not find any methodological flaws in our study or data analysis and simply has different opinion on the meaning of just two terms used in the manuscript. Also, in the first review no problem with the definition of the terms was mentioned by the Reviewer; nevertheless, the terms have been precisely defined in the revised version and should not bring any doubts about their meaning in the study.
