# Supplementary material for: Homogeneity of agriculture landscape promotes insecticide resistance in the ground beetle Poecilus cupreus
Source: PLoS One. 2022 Apr 26;17(4):e0266453. doi: 10.1371/journal.pone.0266453 (PMC9041758; doi:10.1371/journal.pone.0266453)
Supplement: Attachment — Submitted filename: Response to reviewers.docx [file pone.0266453.s002.docx]

**Response to Reviewer and Editor**

We thank the Referee and Editor for the helpful comments. Below we provide point-by-point answers to the points raised by the Reviewer #1 and the Editor.

**REVIEWER #1:**

*1. L27 – Please, ‘inheritable’ should be deleted from the sentence. Even though the sensitivity of P. cupreus was lower in the subsequent generation the inheritability of resistance of P. cupreus to Thiacloprid + Deltamethrin is not proved through these experiments and results.*

We admit that we have not proven that any inheritable resistance mechanism has developed in the studied population, another possibility being the selection of more resistant individuals from the large populations inhabiting the studied areas. We have clarified this in the revised manuscript as follows: “The persistence of reduced sensitivity to Proteus 110 OD for two consecutive generations indicates that either the beetles have developed resistance to the insecticide or the chronic exposure to pesticides has led to the selection of more resistant individuals naturally present in the studied populations” (lines 23-26 in the revised paper without tracked changes).

*2. L80 – Please, see the references Rodrigues et al. 2013 Response of different populations of seven lady beetle species to lambda-cyhalothrin with a record of resistance https://doi.org/10.1016/j.ecoenv.2013.06.014 and Torres et al. 2015 Lambda-Cyhalothrin Resistance in the Lady Beetle Eriopis connexa (Coleoptera: Coccinellidae) Confers Tolerance to Other Pyrethroids https://doi.org/10.1093/jee/tou035.*

We are grateful to the Reviewer for pointing on these important publications, which are now cited in the revised manuscript (line 80 and 391) and have been added to the list of references (lines 549-555).

*3. L181, 183, … – Please, the scientific name of species starts the sentence with full name. This should be corrected in the entire manuscript.*

We have changed our wording across the manuscript as indicated by the Reviewer.

*4. L173 to 188 – The subsection is interesting from the point of view of details on the species studied. However, this subsection contains a lot of information that would not be appropriate for the materials and methods sections. Some of this information can be moved to the discussion section. Information about the species in previous studies detailed here will be important to explain the results found in this research.*

We thought through this comment carefully, but would prefer to leave this sub-section as it was in the original version of the manuscript. In this sub-section, only information which allowed us to select this particular species for our research, determine the time of beetles collecting and select specific research sites (and crop) has been included (e.g., species numerous enough at all studies sites, disperse mainly by walking, easy to identify in the field, with a well-tested culturing procedure, one of the most common and dominant carabids found on arable land across Europe, including oilseed rape). Moreover, as the Reviewer stressed information detailed here is important to explain some of our results. We agree, that description of experimental species, its special characteristics and criteria for selection, does not necessary need to be a separate sub-section in manuscripts; the general methodological approach, including species description, often is a part of Introduction. We are not aware of any strict rules for placing the criteria for selecting species in the manuscript, but we feel it is better to have one consistent paragraph instead of covering species selection criteria in several places in the manuscript. Of course, if the Editor decides that this sub-section must be changed, we see no problem to include some information about the species in other places in in the manuscript, although Introduction seems to be better place at least for some information than Discussion.

*5. L208 – Please, the commercial name of insecticides should be avoided in the manuscript because the commercial name of insecticide could be different in other countries. Instead, the name of insecticide active ingredients would be indicated to use in the manuscript.*

The reviewer is right that reporting the commercial name of an insecticide does not guarantee exactly same composition in different countries, but in this case we cannot refer to the active ingredients only because the purpose of the experiment was to test the actual product used by farmers (i.e., active ingredients + additives), not the specific active ingredients. Please note, however, that the exact contents of the active ingredients in the product are reported in the manuscript (line 218-219). Moreover, although exact concentrations of all product ingredients except the insecticides themselves may differ slightly, the commercial name usually means a mixture of specific chemicals. Hence, we prefer to keep in the manuscript the commercial name of the product used.

*6. L212 to 215 – The mode of action of each active ingredient (Thiacloprid + Deltamethrin) of insecticide (Proteus 110) should not be described in the materials and methods section. This type of information should be suppressed or moved to the introduction or discussion sections.*

As suggested by the Reviewer, the sentence describing the modes of action of both active ingredients has been moved to the Discussion. We also added a few sentences stressing the necessity of using actual formulations in pesticide testing. The section reads now: “Proteus 110 OD is the plant protection product containing two active ingredients, thiacloprid which belongs to neonicotinoids and affects insect nervous system by stimulating nicotinic acetylcholine receptors, and deltamethrin which is a pyrethroid preventing the closure of the voltage-gated sodium channels in the axonal membranes resulting in dysfunction of spiracles – insect death is caused by overdrying. However, the ultimate effect of commercial formulations depends not only on the active ingredients but also adjuvants/co-formulants, which may affect the efficacy of penetration of the active ingredients into insect bodies or the persistence of an insecticide in field conditions [45]. Hence, other formulations with exactly same active ingredients may have somewhat different effects than those reported herein. (lines 369-377).

*7. L215 to L220 – The reasons about the application history of specific insecticides presented here are interesting, but they are not suitable for the materials and methods section. Please, the sentences should be moved to the discussion section.*

The indicated sentence has been moved to the Discussion section (lines 377-380).

*8. L236 to 239 – The sentence should be deleted. The criteria for the assessment of beetles have already been defined in the previous sentence.*

Done.

*9. L247 to 249 – Please, the sentence should be deleted from the statistical analysis subsection. Perhaps, the reference should be left in the sentence. However, the explanation why one was chosen than the other analysis is not appropriate in the materials and methods section. Although this information clarifies why one analysis is appropriate than the other it became a long manuscript with an excess of information.*

Although indeed this sentence may appear not crucial as this can be considered a general knowledge of statistics, the Reviewer admitted himself that “this information clarifies why one analysis is [more] appropriate than the other”. Because we are convinced that in this particular case it is important to clarify why this specific test was used, we prefer to keep this sentence in the manuscript. These are just two lines of text so we don’t think that shortening the manuscript by 30 words is worth sacrificing this information. If, however, the Editor and the Reviewer decide otherwise, we can, of course, make the necessary correction.

***FIGURES***

*Figure 4 – The legend of the x-axis was missed in figure 4. Please, the legend of the x-axis should be inserted in figure 4.*

The x-axis legend has been added (“Habitat type”) and we have also unified with other figures the descriptions of the three plots relating to the three consecutive generations (red uppercase letters “P”, “F1”, “F2”).

**Response to Editor**

*1. Please ensure that your manuscript meets PLOS ONE's style requirements, including those for file naming. The PLOS ONE style templates can be found at*

*https://journals.plos.org/plosone/s/file?id=wjVg/PLOSOne_formatting_sample_main_body.pdf and*

[*https://journals.plos.org/plosone/s/file?id=ba62/PLOSOne_formatting_sample_title_authors_affiliations.pdf*](https://journals.plos.org/plosone/s/file?id=ba62/PLOSOne_formatting_sample_title_authors_affiliations.pdf)

The manuscript has been revised to meet PLOS ONES’s style requirements as provided in the above mentioned templates.

*2. In your Methods section, please provide additional location information, including geographic coordinates of your field collection site if available.*

Information on the coordinates of all twelve study sites has been included as Table 2.

*3. In your Methods section, please provide additional information regarding the permits you obtained for the work. Please ensure you have included the full name of the authority that approved the field site access and, if no permits were required, a brief statement explaining why.*

The explanation of the permits obtained has been added and the text now reads: “The authorisation for capture, transport and keeping the beetles was granted by the Regional Directorate for Environmental Protection in Kraków, Poland, document no. OP-I.6401.128.2017.MMr, and by the Regional Directorate for Environmental Protection in Poznań, Poland, document no. WPN-II.6401.83.2017.AG.2.” (lines 198-201).

*4.* *We note that you have stated that you will provide repository information for your data at acceptance. Should your manuscript be accepted for publication, we will hold it until you provide the relevant accession numbers or DOIs necessary to access your data. If you wish to make changes to your Data Availability statement, please describe these changes in your cover letter and we will update your Data Availability statement to reflect the information you provide.*

Yes, we confirm that repository information for our data will be provided upon acceptance of our manuscript

*5. We note that Figure 1 in your submission contain [map/satellite] images which may be copyrighted. All PLOS content is published under the Creative Commons Attribution License (CC BY 4.0), which means that the manuscript, images, and Supporting Information files will be freely available online, and any third party is permitted to access, download, copy, distribute, and use these materials in any way, even commercially, with proper attribution. For these reasons, we cannot publish previously copyrighted maps or satellite images created using proprietary data, such as Google software (Google Maps, Street View, and Earth). For more information, see our copyright guidelines:* [*http://journals.plos.org/plosone/s/licenses-and-copyright*](http://journals.plos.org/plosone/s/licenses-and-copyright)*.*

The above mentioned figure is neither in whole nor in part a satellite image. The upper part of the figure showing location of the two study landscapes in the Wielkopolska province and the location of the Wielkopolska province in Poland was generated using data from the National Register of Boundaries in Poland which is provided free of charge (via the service [www.geoportal.gov.pl](http://www.geoportal.gov.pl)) and can be used for any purpose according to the amendments to the Surveying and Cartographic Act from April 16 2020. The land-cover maps of our study landscapes shown at the bottom of the figure are simplified versions of very detailed land cover maps generated by us in a step-by step process using the methodology described in Ziółkowska et al. (2021) based on two types of source data: (1) the National Database of Topographic Objects (BDOT) providing the land cover and land use information at the scale of 1:10.000, and (2) the Land Parcel Identification System (LPIS) providing information on the type of cultivated crops (see Methods section). We are the copyright holders of these products. However, the source data (BDOT database and data on parcels - Land and building register) are also provided free of charge (via the service [www.geoportal.gov.pl](http://www.geoportal.gov.pl)) and can be used for any purpose according to the amendments to the Surveying and Cartographic Act from April 16 2020. Therefore, the entire figure may be published under the Creative Commons Attribution License (CC BY 4.0).

In addition, to be more clear, we have now added the following explanation to the main text of the manuscript: “Raster land-cover maps (resolution of 1 m^2^) for the landscapes were generated in a step-by-step process according to the methodology described in [33] by combining data from: (1) the National Database of Topographic Objects (BDOT) providing the land cover and land use information at the scale of 1:10.000, and (2) the Land Parcel Identification System (LPIS) providing information on the type of cultivated crops.” [lines 145-149]

We also provided some additional explanation in the Figure 1 caption:

“Land-cover maps were generated according to methodology described by Ziółkowska et al. [33], see Methods section for more details.” [lines 139-140].
