# Supplementary material for: Homogeneity of agriculture landscape promotes insecticide resistance in the ground beetle Poecilus cupreus
Source: PLoS One. 2022 Apr 26;17(4):e0266453. doi: 10.1371/journal.pone.0266453 (PMC9041758; doi:10.1371/journal.pone.0266453)
Supplement: Attachment — Submitted filename: Response to reviewers.docx [file pone.0266453.s003.docx]

**Response to Reviewer**

We thank the Referee for the helpful comments. Below we provide point-by-point answers to the points raised by the Reviewer #1.

**REVIEWER #1:**

*1. L19, 24, 105, 119, 214, 227, 235, 284, 292, 314, 326, 362, 367, 369, and 402 - The reason given in the section of the response to the reviewers by the author for keeping the insecticide trade name Proteus 110 OD in the manuscript is conflicting because the insecticide Proteus 110 OD is a mixture of ingredient actives thiacloprid + deltamethrin. Several products with ingredient actives mixture have been released in the last years. What were the additives tested in the experiment? Oil dispersion (nonpolar) allows break plant barriers such as the wax layer of leaves plants (nonpolar) to lead the ingredient active improving an insecticide’s uptake by the plant. Acetone is an adjuvant that should not have to act in the beetle. The suggestion to change the trade name of Proteus 110 OD to its active ingredient was based on making it internationally standardized to identify it other countries exclusively through the name of ingredient active. However, decisions can be made by consensus of the author and editor.*

We still agree with the Reviewer on the fact that when reporting the commercial name of an insecticide, one can expect different composition in different countries. However, we stand by our arguments that providing information only on active ingredients, without reporting commercial name, may be confusing. By giving the full name of the product, together with the exact contents of the active ingredients in the product and the country of purchase (lines 212-218 in the revised paper without tracked changes), we avoid any doubts whether the toxicity of the active ingredients alone could be potentially different from that of the mixture (i.e. the commercially available product). The purpose of the experiment was to test the actual product used by farmers (i.e., active ingredients + additives), not the specific active ingredients, as we wanted to reflect as closely as possible the real situation in the field. To make it unambiguously clear what the composition of the product used was, we have included, as Supporting information, a table with product specification as provided by the manufacturer (Bayer, Germany) (S1 Table). Additional text in lines 217-218 in the revised manuscript was also added: “(see Table S1 in Supplementary materials for full product specification)”.

*2. L81 to 82, 86, 95, 171, 236, 268, 269, 352, 395, 397, 421, 465 and 470 – Please, all references used in the sentence referred to insecticides. Thus, pesticides should be changed to insecticides. Pesticide is a broad term that includes various products to kill different types of organisms, insecticide is a term designated for products that act specifically on insects. Please, the correct term should be reviewed in the entire manuscript.*

Of course we agree with the Reviewer that pesticide is a broad term that includes various products (e.g. insecticides, herbicides, fungicides) to kill different types of organisms, whereas insecticides act specifically on insects. We have now changed the term “pesticide” into “insecticide” wherever we refer to our laboratory test results on Proteus 110 OD, which is the insecticide (lines 236, 268, 269, 352, now lines 234, 265, 266, 345). The changes were also done in lines 81-82 and 86 (now lines 79-80 and 84): although the references we used are not specific to insecticides (they relate to resistance to xenobiotics in general), we agree that for consistency with the earlier sentence, in which “insecticides” were mentioned, the same term should be used in the indicated sentences. In line 94, the term “pesticide” was mentioned because the use of other products then insecticides (e.g. herbicides, fungicides), even though they are not directly harmful to ground beetles, still may indirectly influence their survival, for example through the impact on their habitat and/or food source (e.g., disappearance of plant food). Therefore, the use of the term “pesticide” is appropriate in this context. A similar situation arises in line 171 (now line 169 in the revised paper without tracked changes) where the term “pesticide” is used to emphasize that the populations of the two habitats mentioned (S0 and L0) were not exposed to any potentially toxic substances in the field. The use of the term “insecticides” would imply that other types of treatments could have been applied there. A similar justification for using a term “pesticide” applies to line 421 (now line 411). Regarding lines 465 and 470 (now lines 455 and 460), we decided to keep the original terminology, as we draw general conclusions here on either significance of landscape for insect response (not resistance) to pressures from different pesticides used in that landscape (line 465, now line 455) or resistance of beneficial species (not specifically insects) to pesticides (line 470, now line 460).

*3. L345 to 348 – The broad confidence intervals to the LT50 values for insecticide-treated beetles from the LM and LL habitat types and acetone-treated beetles from the control habitats (S0-A and L0-A, Fig 4) should be discussed in the discussion section. Why were only beetles from these areas had a broad confidence interval in the different generations' studies (P, F1, and F2)?*

The confidence intervals to the LT50 were calculated fusing Litchfield’s method (lines 256 – 258 in the previous version of the manuscript) which is a method for rapid graphic solution of time-per cent effect curves. We have now checked that confidence intervals are much narrower when they are calculated for medians (LT50 is the median lethal time). To give an example, for insecticide-treated beetles from LL habitat types in F2 generation, with LT50 of 15.23 days, the confidence interval calculated using Litchfield’s method is between 5.67 and 40.92 days, whereas the confidence interval for the median of 15.23 days is between 12.23 and 16.23 days (based on equations on <https://www.statology.org/confidence-interval-for-median/>).

However, following the Reviewer’s remark about the broad confidence intervals to the LT50 values we concluded that we unnecessarily confused the reader suggesting that confidence intervals were used to compare the mortality rates between the habitat types. In fact, as stressed in table 3, we compared the whole survival curves using Wilcoxon test (“Groups with the same lowercase letter do not differ significantly at p ≤ 0.05 in terms of survival (pairwise comparison of survival curves, Wilcoxon test)”, now lines 299). Because of that, we decided to replace the Figure 4 with a similar figure, but instead confidence intervals, standard errors for LT50 values are now presented. Thus, all sentences refereeing to confidence intervals in Statistical analysis and results describing differences comparison of LT50s between habitat types based on confidence intervals overlap have been removed from the revised version of the manuscript.

*4. L339 and 351 - How was it determined that the beetle was tolerant to insecticides? Would not this be an evolution of the beetle's resistance to insecticides? The term used to describe the results found in the experiment should be clearer.*

Each individual beetle from each habitat type had its own survival time after insecticide treatment (i.e., its own insecticide sensitivity = insecticide tolerance) and we showed that: “as in the P generation, the comparison among insecticide-treated beetles from different habitat types revealed statistically significant difference in survival rates of beetles in both the F1 and F2 generations (p < 0.001 for F1, p < 0.03 for F2, Fig 3)” (now lines 332 - 335) and “The increased tolerance to the insecticide found in beetles from the P generation from the LM and LL habitats was still present in both next generations F1 and F2, as insecticide-treated beetles from the LM and LL habitats survived significantly better than insecticide-treated beetles from all other habitat types (Fig 3, Table 3), but significantly worse than acetone-treated beetles from the control habitats (S0-A and L0-A; Table 3)”. Thus, because similar results of increased tolerance to the insecticide were found in beetles from the LM and LL habitats over three generations (P, F1 and F2), the results suggest the evolution of resistance. Results and Discussion sections are, however, separated in the manuscript, so we do not interpret our results in Results section. We assumed that the Discussion section is designed to tell what the results mean. Thus, we would prefer to keep our original writing and first present the results on increased tolerance to the insecticide in beetles from the LM and LL habitats over three generations (P, F1 and F2) and then conclude that such results suggest the evolution of resistance. Nevertheless, if the Editor decides otherwise, we don't see a problem with adding following summary/conclusion sentence to the Results section: “Thus, the results suggest that the effect of insecticides on the evolution of resistance in populations of *P. cupreus* strongly depends on large-scale landscape characteristics.”.

*5. L360 to 362 - The first sentence of the discussion section contradicts the results described in the materials and methods section, see comments above in lines 339 and 351. In materials and methods, the tolerance of the beetle to insecticides has been described. However, the discussion concerns the development of insecticide resistance in the beetle. Please, the terms used in the materials and methods section and the discussion should be clearer.*

Yes, in the Material and Methods section we described the methods used to study the tolerance of the beetles to insecticide (in other words, we studied susceptibility of beetles to pesticide). This tolerance was studied for three generations to sort out possible temporary effects through direct selection of the most resistant individuals collected from the field right after the spraying from parental effect and/or possible genetically fixed resistance to insecticides (as stressed already in the Introduction, lines 114 -118 in the revised paper without tracked changes). As we stressed in the Discussion section, the successful culturing of the beetles through three generations enabled us to separate the actual genetically fixed adaptation from possible direct or maternal effects (lines 443 - 445). Thus, we don’t see any contradiction between Methods (testing tolerance of beetles to insecticides in generation P, F1 and F2) and Discussion sections (drawing conclusions from obtaining similar results on tolerance of beetles to insecticide in all three generations). Please see also our answer to the previous comment as well as to the last comment.

*6. L377 to 380 - The sentence is confusing as a massive number of studies have used both commercial formulations and unique active ingredients. Please, the sentence should be rephrased or deleted from the discussion section.*

As suggested the mentioned sentence has been deleted form the discussion section.

*7. L408 to 409 – There was no found tolerance as well as the use of pesticides in this study mentioned in the sentence. Schoonees and Giliomee (1982) found an increase of resistance of two strains of parasitoids to the insecticides in different localities where the application of insecticide was intensified through the years.*

Schoonees and Giliomee (1982) evaluated the toxicity of methidathion (organophosphorus insecticide) to two strains of *Aphytis africanus* and *Comperiella bifasciata*, both parasitoids of *Aonidiella aurantii* in South Africa. They found that different geographic strains varied; one of them, which used to receive three to four sprays of organophosphorus insecticides per year over many years, was much more tolerant to methidathion than the one, which received only one spray per year. To be precise, the authors used the word “susceptibility” to check difference of the studied species between localities, but being more susceptible to something means less tolerance to it. In the Methods they described how this “susceptibility” was measured and expressed (survival was checked in contact toxicity test and LC50 was calculated). In the Results they described differences in susceptibility between the two strains (collected from different localities) and calculated “ resistance factor”, which is LC50 of one strain divided by LC50 of the second strain. In the Discussion section, the authors concluded about “the greater resistance to methidathion in *A. africanus* and C. *bifasciata* from Letsitele as compared with those from Mooinooi and Zebediela” which simply means that *A. africanus* and *C. bifasciata* from Letsitele had higher LC50 (were less susceptible to methidathion = were more tolerant to methidathion) as compared with those from Mooinooi and Zebediela.

The paper by Schoonees and Giliomee (1982) proves that organisms subjected to higher number of insecticide sprays are able to develop tolerance to the most commonly used product (=are more resistant, and the word “tolerance” has been now replaced by the “resistance”). We agree that the paper does not directly answer the question of whether landscape structure is one of the causes of resistance (=being more tolerant to methidathion). However, the described situation (higher number of sprays) may occur more often in homogenised landscapes where the insects inhabiting them have limited or no access to a refuges. With this in mind, we consider the work by Schoonees and Giliomee (1982) as a good example that more frequent contact with insecticides promotes the development of resistance.

***ADDITIONAL COMMENT***

In Table 3, we made a typo error for the LT50 values of beetles from P generation and LM habitat. Instead of 21.25 it should be 23.26.
